# Delivering systems-level change to improve post-diagnostic dementia support: Qualitative findings from the PriDem study

**DOI:** 10.1371/journal.pone.0317811

**Published:** 2025-03-28

**Authors:** Emily Spencer, Katie Flanagan, Marie Poole, Federica D’Andrea, Maud Hevink, Jane Wilcock, Kate Walters, Louise Robinson, Greta Rait, Sarah Griffiths, on behalf of the PriDem Study project team

**Affiliations:** 1 Research Department of Primary Care and Population Health, Institute of Epidemiology & Healthcare, Faculty of Population Health Sciences, University College London, London, United Kingdom; 2 The Marie Curie Palliative Care Research Department, Division of Psychiatry, University College London, London, United Kingdom; 3 Faculty of Medical Sciences, Population Health Sciences Institute, Campus for Ageing and Vitality, Newcastle University, Newcastle upon Tyne, United Kingdom; 4 The Gellar Institute of Ageing and Memory, School of Medicine and Biosciences, University of West London, London, United Kingdom; 5 Department of Psychiatry and Neuropsychology, School for Mental Health and Neuroscience, Alzheimer Center Limburg, Maastricht, The Netherlands; Erasmus University Rotterdam, NETHERLANDS, KINGDOM OF THE

## Abstract

**Background:**

There are 900,000 people with dementia in England and Wales. Existing models of post-diagnostic support are unsustainable and unaffordable. The PriDem programme developed a new model of primary care-based dementia care, whereby a Clinical Dementia Lead (CDL) would facilitate systems-level change.

**Aim:**

To assess barriers and facilitators to implementation of the PriDem intervention.

**Methods:**

7 general practices participated in a qualitative process evaluation, as part of the mixed-methods feasibility and implementation study. Practices were located within 4 Primary Care Networks in the North East and South East of England. 26 healthcare professionals, 14 people with dementia and 16 carers linked to participating general practices participated in semi-structured individual and small group interviews. Additional qualitative data were generated through nonparticipant observations and researcher fieldnotes from CDL intervention supervision sessions. Data were analysed using abductive codebook thematic analysis informed by Normalisation Process Theory (NPT).

**Results:**

Six themes were generated: 1) The rocky ground of primary care; 2) The power of people; 3) Tension between adaptability and fidelity; 4) Challenging the status quo: reimagining care planning; 5) One size doesn’t fit all; 6) Positive effects on people and systems: towards sustainability. Through the lens of NPT we can understand the contextual challenges facing primary care, the mechanisms (e.g., work undertaken by individuals) to overcome those challenges, as well as the potential outcomes of such an approach, in terms of longer-term sustainability of changes made.

**Conclusions:**

Despite the pressures facing primary care within England and Wales, meaningful change can be made to practice in the care of people with dementia. The presence of motivated and engaged staff are critical to implementation, as is ensuring understanding of complex interventions, so that fidelity can be maintained. People with dementia and carers benefitted from improved care systems. Commissioners should consider the benefits of a CDL-led approach.

## Introduction

Dementia is a syndrome affecting cognitive functioning, leading to changes in emotions, behaviour, communication, and difficulty performing activities of daily living [1]. In England and Wales, 900,000 people live with dementia. As a result of population ageing, this figure is projected to rise to 1.7 million by 2040 [2]. Over the same period, the total costs of UK dementia care are expected to increase from £34.7 billion to £94.1 billion per year [3]. Following diagnosis, care is often inadequate and poorly integrated across health and social services [4,5]; around half of people with dementia report receiving insufficient support [6]. Policy and research emphasise the importance of improving the quality and accessibility of care [7–11]. The 2016 World Alzheimer Report highlighted that existing specialist-led models of post-diagnostic care, situated within secondary care, are unsustainable and unaffordable [12]. Instead, a more efficient use of existing resources could be achieved via the introduction of a task-shifted and task-shared approach, in which primary care takes lead responsibility for post-diagnostic care coordination, facilitating appropriate specialist care input as needed. Such an approach may lead to improved continuity of care, more holistic support, as well as a reduction in stigma for people with dementia [13].

Although barriers to such an approach have been identified, including limited capacity and capability of generalist staff [11], research suggests that both the feasibility and acceptability of primary care-led dementia care can be improved through strengthening joint working, supporting non-specialists, and the introduction of dementia-focused staff into primary care [8,11].

### The PriDem programme

PriDem (Primary care led post-diagnostic Dementia care) (2019-2023) was a research programme which aimed to develop a feasible and acceptable evidence-based model of care. In line with the recommendations of the 2016 World Alzheimer Report [12], this model would involve primary care teams coordinating post-diagnostic dementia care. The programme comprised five workstreams, of which this paper reports on workstream four. In the first two workstreams, existing primary care-led models of dementia care and long-term conditions were examined [8,14,15], alongside qualitative interviews to identify proposed models of best practice [13,16].

Unique to this programme, during workstream three a manualised intervention was co-created with a patient, public and professional stakeholder group [17]. The intervention was designed to be facilitated by a Clinical Dementia Lead (CDL), a specialist nurse or allied health professional, focusing on three interlinking strands [18]:

1. **Developing systems** for delivery of evidence-based, post-diagnostic support. CDLs would work with local stakeholders to review referral and transition processes, develop a map of local dementia services, and establish a named point of contact for each person with dementia.
2. **Delivering tailored care and support** to people with dementia and their carers. CDLs would work with practice teams to optimise annual dementia reviews and deliver personalised care planning, as well as providing advice and management for people with more complex needs.
3. **Building capacity and capability** by supporting non-specialists to deliver multi-disciplinary post-diagnostic care and upskilling staff through support and training. CDLs would work with staff to develop practice-based dementia teams.

Through these strands, CDLs would engage with general practices within Primary Care Networks (PCNs; groups of general practices and other health and social care organisations collaborating to deliver integrated services) to deliver comprehensive, systems-level change. The intervention was designed to be delivered flexibly, in order to best meet the needs of general practices, considering variability in existing resources and expertise.

In workstream four, the PriDem intervention was evaluated through a 15-month mixed-methods feasibility and implementation study. The intervention aimed to maintain and improve quality of life for people with dementia and their carers, measured by a range of dementia-specific self-report questionnaires. Details of quality of life measures and quantitative findings are reported elsewhere [19]. We have previously reported that the intervention resulted in improved provision of personalised care planning at participating general practices, assessed quantitatively through an audit of electronic medical records [20]. Systems-level change made through the intervention were considered to be feasible and acceptable within the context of primary care [19]. This paper reports on the findings of the qualitative process evaluation. Process evaluations examine implementation (processes, quality and quantity), mechanisms of impact (how interventions trigger change) and contextual factors influencing delivery of an intervention [21]; i.e., exploring *how* interventions are implemented in practise, and how this may influence impact.

### Aim

To assess barriers and facilitators to implementation of a primary care model of post-diagnostic support (the PriDem intervention).

### Objectives

- To examine how the intervention is delivered and adapted within practice
- To identify context and delivery variations/factors which influence embedding the intervention in usual care
- To identify factors that increase adoption, coverage and sustainability of the intervention including acceptability, appropriateness, fidelity
- To explore context, mechanisms and impact of the intervention for people with dementia, carers and professionals

## Methods

### Study design

A mixed-methods feasibility and implementation study was conducted between February 2022 and June 2023, including a qualitative process evaluation. This process evaluation considered the perspectives and experiences of health and social care professionals, people with dementia and carers to examine barriers and facilitators to successful implementation of the PriDem intervention. Normalisation Process Theory (NPT) [22] was used as an underpinning theory throughout, including in study design and data analysis. NPT offers a framework with which to examine the work undertaken, individually and collectively, to implement an intervention, considering the *contexts*, *mechanisms* and *outcomes* at play. The study protocol has been published [17].

### Ethical consideration

Ethical approval for the study was obtained from Wales REC 4 of the National Research Ethics Service (21/WA/0267). Confidentiality Advisory Group (21/CAG/0182) recommended that support under Regulation 5 of the Health Service (control of patient information) Regulations 2002 (‘section 251 support’) was given for the processing of patient information, enabling researchers to access specific patient data without prior informed consent. Written or verbal informed consent was obtained for all study participants.

### Setting

Seven general practices from four PCNs participated. Four practices from three PCNs were located in the North East, with three practices located within one PCN in the South East of England. A CDL was recruited for each region to deliver the intervention over 12 months. CDLs underwent training delivered by the research team, received clinical supervision with a specialist dementia nurse and intervention supervision with members of the research team.

### Participants

Researchers and CDLs collaborated to identify professionals for interview and observation opportunities. We aimed to interview up to 28 professionals, including general practice staff, external staff and commissioners of local dementia services. CDLs and their clinical supervisor were also invited to participate.

We aimed to complete 14 observations, including interview participants as well as professionals who had not been interviewed.

People with dementia and carers were recruited from the feasibility and implementation study. People with dementia were eligible for inclusion in interviews where they had capacity to consent to participation. We aimed to interview up to 20 people with dementia and up to 20 carers. These sample sizes would allow for collection of enough rich data, across a diverse sample, with sufficient ‘information power’ [23] to address the research aims.

Inclusion and exclusion criteria are presented in Table 1. Recruitment for the process evaluation took place between 09/06/2022 and 18/07/2023. Details of the recruitment process are reported elsewhere [17].

**Table 1:** Study inclusion and exclusion criteria.

Participant group	Inclusion criteria	Exclusion criteria
Professionals (health and social care professionals and commissioners)	Over 18 years old; working for or with people with dementia in participating sites; willing and able to provide informed consent	Do not provide or commission post-diagnostic dementia support
People with dementia	Over 18 years old; registered with a participating general practice; diagnosis of dementia recorded in medical record; community dwelling; capacity to consent to the study	Judged as inappropriate for the study by a member of the primary care team (e.g., due to concurrent life events such as bereavement or receiving end-of-life care); patients with an advance statement indicating they do not wish to take part in research studies; living in a care home
Carers	Over 18 years old; carer of a person with dementia who has agreed to take part in the feasibility and implementation study; willing and able to provide informed consent	Judged as inappropriate for the study by a member of the primary care team (for the same reasons as the person with dementia); non-fluent English speaking

### Data collection

Data collection was undertaken by a trained team of researchers with collective expertise in qualitative methods and dementia research. Junior members of the research team received supervision and feedback from more experienced members. In one site, a senior researcher had previous interactions with professional participants through prior work. Other than this, researchers and participants met through the course of the PriDem study. Where possible, people with dementia and carers were interviewed by the researcher with whom they had interacted during the study.

Individual or small-group semi-structured interviews were conducted face-to-face or online via videoconferencing software, using a topic guide. With healthcare professionals’ interviews, topic guides explored intervention acceptability and feasibility, alongside implementation barriers and facilitators. For people with dementia and carers, questions focused on direct exposure to the intervention, as well as perceived changes within the general practice (indirect exposure). Topic guides were informed by NPT [22], with questions designed to explore intervention *contexts*, *mechanisms* and *outcomes*. These were iteratively revised to include areas of importance identified during previous interviews [24], as well as to examine specific innovations arising over the course of the intervention (e.g., dementia one-stop-shops, a novel approach to care planning developed after the start of data collection). Interviews were audio-recorded, transcribed verbatim, checked and anonymised.

Researchers undertook nonparticipant observations [25] of non-clinical intervention activities conducted by CDLs, such as multi-disciplinary team (MDT) meetings and training sessions. Observations took place in-person or online, with researchers producing detailed fieldnotes, informed but not constrained by NPT implementation constructs.

Researcher fieldnotes were generated following interviews, as well as from the content of CDL intervention supervision meetings, providing additional contextual detail for analysis.

### Data analysis

An abductive, codebook approach to thematic analysis [26] combined inductive coding with theorising using NPT [22].

Three researchers (Authors 1, 4 and 5) undertook an initial line-by-line coding of early data. Different data sources were coded: transcripts of interviews with professionals, people with dementia and carers, and fieldnotes from observations and intervention supervision. Following team discussion of these early, inductive codes, an initial codebook was developed by Author 1 and Author 2. As new data were coded, codes were combined, abandoned or formulated to create the final codebook. The codebook was applied across the entire dataset by Author 1 and Author 2. Author 10 resolved coding disagreements, reviewing a selection of codes to check for consistency of analytic process, aiming for a ‘stable perspective’ in codebook application [27].

Themes were developed by Author 1 and Author 2, in discussion with the wider research team, through the grouping of codes according to patterns of meaning that directly addressed the research questions.

Finally, NPT was used to make sense of the findings. As the primary focus of NPT is those delivering the intervention, the theory was used only with data from interviews with professionals, observation and intervention supervision fieldnotes. Relevant themes were mapped against NPT constructs and subconstructs in order to create an understanding of the processes underlying implementation.

## Findings

### Participant demographics

28 interviews were conducted with 26 healthcare professionals, mean length 34 minutes (range 19-80 minutes). 26 were individual interviews, two included two participants each. Four participants took part in a follow-up interview focusing on intervention sustainability.

14 people with dementia and 16 carers participated in a total of 21 interviews, of which nine were dyadic (i.e., involving the person with dementia and their nominated carer). Interviews lasted a mean of 34 minutes (range 18-67 minutes).

Participant demographics are presented in Table 2.

**Table 2:** Interview participants’ demographic data.

	Professionals (n = 26)	People with dementia (n = 14)	Carers (n = 16)
**Age**			
25-35	7		
36-45	4		
46-55	10		3
56-65	3		3
66-75		4	3
76-85		9	5
86-95		1	2
Missing	2		
**Sex**			
Male	3	10	1
Female	21	4	15
Missing	2		
**Ethnicity**			
White	18	12	12
South Asian/East Asian/Asian British	4		1
Black/African/Caribbean	2	1	1
Other Ethnic Group		1	2
Missing	2		
**Professional role**			
GP	9		
Social prescriber	4		
Practice manager	2		
Care coordinator	2		
Third sector	2		
Commissioner	2		
CDL	2		
CDL clinical supervisor	1		
Dementia advisor	1		
Operations manager	1		
**Dementia type**			
Alzheimer’s		10	
Mixed		3	
Lewy Body		1	
**Marital status**			
Married		8	12
Widowed		3	
Divorced		2	2
Separated			2
Single		1	
**Living status**			
With spouse		8	
Alone		4	
With other family		2	
**Relationship to person with dementia**			
Spouse			11
Sibling			2
Son/daughter			2
Friend			1

Fourteen nonparticipant observations were conducted; participants’ demographic data were not collected.

### Thematic analysis findings

**Fig 1:**
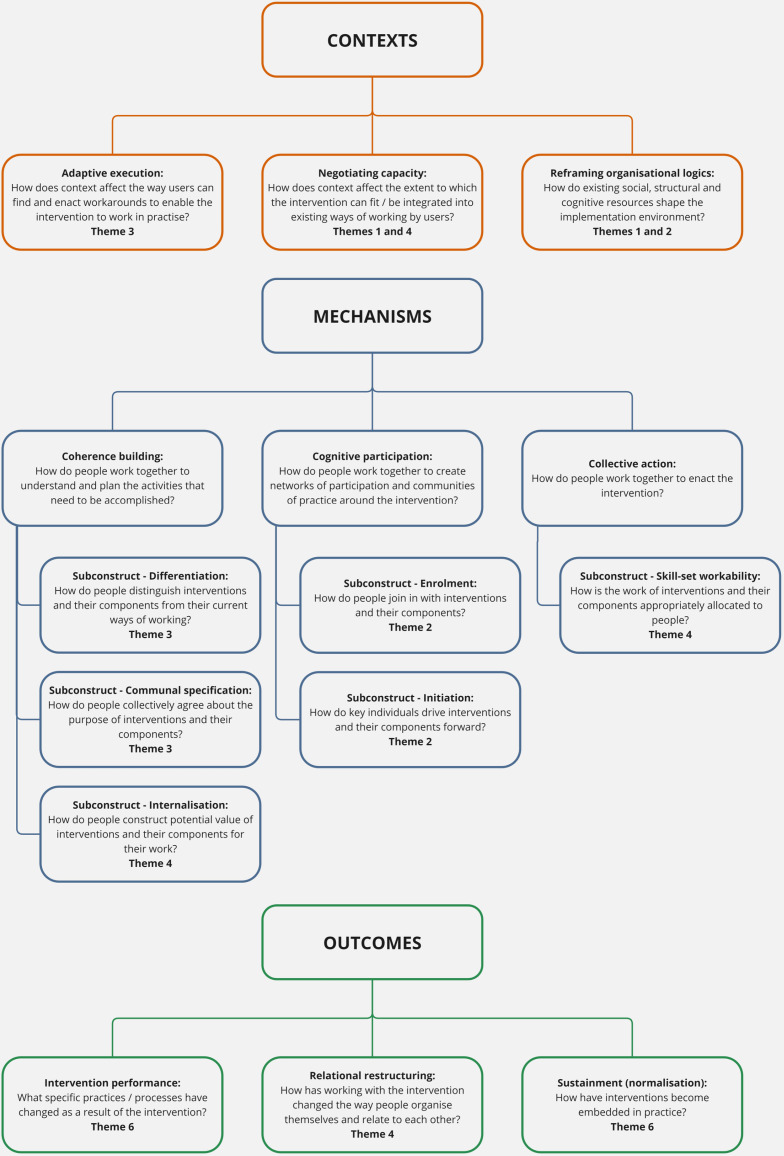
Normalisation process theory constructs and subconstructs made relevant through thematic analysis. Based on May et al., 2022 [22].

A subset of NPT constructs became relevant during analysis, see Fig 1. Six interacting themes were developed, considering the key facilitators and barriers to implementation. Themes are presented with illustrative quotes, linking to NPT where appropriate.

#### Theme 1: The rocky ground of primary care.

Time and workload constraints were common issues facing primary care staff, particularly in the post-COVID recovery context. For some, this served as a barrier to engagement with the intervention, particularly in the early stages of implementation when it was difficult to gauge potential benefits. Increased hybrid working introduced an additional challenge for CDLs when working to engage staff.

*…there’s an environment at the moment where it’s very difficult to facilitate change because everybody is exhausted after the pandemic […] they’ve got so many challenges ahead of them.* PROF-15 (CDL clinical supervisor)

High staff turnover and a lack of continuity of care was an issue for practices and patients. People with dementia and carers experienced difficulties accessing appointments and struggled to know who to contact or which services were available, an issue exacerbated by the pandemic. Some participants reported variability in access to dementia-specific care or expertise:

*I wouldn’t know who to ask for now. […] So far I’ve not needed them, but when I do need them, I wouldn’t know who to ask for. And I wouldn’t know if they’re any good.* C-05 (carer).

Professionals spoke about financial barriers to provision of high-quality dementia care. Although increased funding was seen by many as a necessary condition for change, there was evidence that even financially incentivised activities, such as care planning reviews, were unfulfilled. Where reviews did take place prior to the PriDem intervention, these were often reported as limited to activities such as blood pressure checks, with no reference to dementia. Many patients had never been offered a dementia review, despite assuming this would form part of usual care:

*I presumed that once [husband] had been diagnosed that he would be followed up periodically, like see how his memory was and how he was coping with those tablets and things like that. But absolute zilch, nothing.* C-15 (carer)

Despite provision of research support costs, a CDL and practical resources to facilitate improved patient care, for some practices the lack of remuneration for intervention activities deterred engagement.

For people with dementia, carers and professionals alike, resourcing constraints within primary care represented rocky ground in which to deliver quality dementia care. This theme can be considered in terms of NPT *Contexts,* where pre-existing and unfolding aspects of the intervention’s environment shape implementation. Existing pressures on staff teams led to challenges in terms of considering how the intervention could be successfully integrated into usual ways of working (*negotiating capacity*), with awareness that limitations in existing resources (*reframing organisational logics*) created additional challenges when considering implementing changes to established practices.

#### Theme 2: The power of people.

CDLs played an integral role in intervention delivery, with their personal attributes identified as key facilitators. Early in the intervention, CDLs developed relationships with staff to promote engagement, working to overcome the contextual constraints identified in Theme 1. CDLs often relied on informal opportunities to make connections, balancing persistence with sensitivity to the pressures facing primary care. An existing awareness of the context was seen as an advantage, promoting engagement with the intervention:

*[CDL is] a dynamo, her character means that you want to engage with her, alright, so anyone that comes in has got to kind of be, I’m not going to say just as vivacious and bubbly, but they need to have a real understanding of what is needed within a [GP] surgery.* PROF-10 (Care coordinator)

Pre-existing dementia interest and knowledge acted as facilitators to engagement, as well as key staff members championing the intervention. CDLs were able to identify influential individuals to help drive implementation. These varied from GPs, who were able to enrol staff and prioritise dementia care across the practice, to junior staff, who promoted change:

*There’s a lot of interest there, from [social prescribers] who, they would like to develop more post diagnostic support for families. So I’ve kind of identified a group of professionals, if you like, who are really keen and motivated.* PROF-04 (CDL)

Upskilling staff was a key intervention component, delivered flexibly according to the needs of practices. There was wide variation in practice engagement: where some practices proactively offered regular education meetings or invited teaching on agreed topics, others declined to participate in formal training. Many staff benefited from informal upskilling, learning through the modelling of work undertaken by CDLs. Despite the flexibility of this component of the intervention, some staff groups, such as GPs, remained difficult to reach.

Practice hierarchies also impacted the CDLs’ ability to access different groups. On occasion, individuals acted as gatekeepers, preventing CDLs from engaging those who could potentially influence the course of the intervention:

*I got the impression that [practice manager] was very protective of the GPs and didn’t want to give the GPs unnecessary work or any unnecessary demands. […] So we would meet and I would make some suggestions, and she would take them back to the meeting, and then come back and say, “Well, they’ve said, ‘No.’” So trying to get past that was really hard. And I mean in the end I directly emailed the particular GP and said, “Could we meet?” […] So in the end I did go around the practice manager.* PROF-04 (CDL)

Individuals were influential in implementation, whether that influence be facilitative or inhibitive. Identifying a local champion was key to successful implementation. This theme can be considered in terms of *Contexts*, and *Mechanisms*; the collaborative work undertaken to achieve implementation goals. Organisational hierarchies were an implementation barrier, however existing knowledge, interest and relationships were harnessed to facilitate change in engaged practices (*reframing organisational logics*). The work of CDLs and key staff can be considered in terms of *cognitive participation*, with engaged participants considering themselves the right individuals to drive implementation (*enrolment*), and actively seeking to engage others (*initiation*).

#### Theme 3: Tension between adaptability and fidelity.

Intervention adaptability and agility was highlighted as a key benefit. Such flexibility allowed staff to innovate solutions to meet the needs of their practice, improving individual buy-in.

*...we wanted to find a way to make everyone conscious that they needed to be aware of our patients that need a little bit more support. So we all sat down and we brainstormed for a bit and I’m not going to say it was just me, but […] I kind of said, “Well why don’t we just use the major alert, it’s the quickest and easiest way for all of us to be aware.”* PROF-10 (Care coordinator)

Practices were given access to evidence-based bespoke resources, for example, comprehensive templates for holistic dementia annual reviews. Staff adapted resources to their context, enhancing their utility:

*…so we looked at the PriDem information and the templates and stuff that [CDL] kind of went through with us and then we cross-matched it with what we use on our computer system […] and so we tried to kind of create a more user-friendly version for ourselves to use at the clinic…* PROF-09 (GP)

While the benefits of flexibility were clear to professionals, on occasion this created tension with maintaining intervention fidelity in terms of the clinical component of the CDL role. Early in the intervention, CDLs used their clinical skills to embed within primary care, gaining trust with clinical staff:

*[CDL] points out that her clinical credibility is working as her passport to engagement – she feels that if this is taken away from her it will be a barrier to engagement with practices. Seeing/discussing patients is a way in/a hook.* Researcher reflections from CDL training

An emphasis on clinical work could, however, lead to a misunderstanding of the intended function of the CDL role. In some cases, practice staff viewed the CDL as an extra staff member for direct patient care delivery, alleviating staff capacity issues seen in Theme 1, rather than as a resource to develop effective systems:

*She’s been seeing patients [...] secretary’s been referring anybody coming through as a new patient, she’s been referring to [CDL] […] any new patients that have needed help and support [CDL’s] dealt with them.* PROF-19 (Practice Manager)

There was variability in terms of patients referred to CDLs, some of which did not meet pre-agreed criteria regarding complexity. One CDL spoke directly of the tension between managing a clinical workload and maintaining intervention fidelity:

*…the GPs just wanted me to run along and do patients, but that wouldn’t be sustainable and wasn’t what the project was about.* PROF-01 (CDL)

The adaptability of the intervention was beneficial for practices, giving individuals ownership over changes, and ensuring the intervention met the needs of the local context. While an increased clinical presence had clear benefits for practices, over-reliance on CDLs performing clinical tasks risked stretching intervention boundaries beyond the point of fidelity, and changes made being unsustainable. This theme can be considered in terms of *Contexts* and *Mechanisms*. While innovation can be seen as an example of *adaptive execution*, issues with *coherence* are apparent for some stakeholders, with some failing to understand the nature of the intervention (*communal specification*), or how it is distinct from other services (*differentiation*).

#### Theme 4: Challenging the status quo: reimagining care planning.

Improvement to dementia care planning was the key aim of the intervention. Supported by CDLs, practices developed and iteratively refined their own care planning processes, with two practices implementing dementia review ‘one-stop-shops’. People with dementia and carers were invited to attend a clinic where they would meet with several members of the MDT. Tasks were organised so that each staff member would have responsibility for specific care domains, leading to a more holistic approach to care planning.

*The idea was that they would be greeted by the Care Coordinator and have their weight, blood pressure, height checked, make sure we had all the details correct and then they would have an individual appointment with [CDL], with [GP], and with our [dementia advisor] who came to the surgery to give the family members some information. So it was kind of a one stop, all-in clinic.* PROF-09 (GP)

Initially, offering such comprehensive reviews was a time-consuming process. However, running a series of clinics over the course of the intervention allowed practices to identify areas for improvement and opportunities to streamline the process, improving the sustainability of these changes:

*So we had to adapt it after the first clinic it just became – it was too overwhelming so we adapted it…* PROF-09 (GP)

In some sites, utilisation of the wider MDT meant that specific patient groups, for example housebound patients, could be offered a service they had previously been unable to access.

Some practices and staff were reluctant to change existing care planning approaches, even when lacking in quality, or questioned the purpose of annual reviews. For some, offering this service appeared inequitable, or required justification:

*I’ve got an interest in dementia, but equally […] is this actually fair, if we did decide to give somebody 20 minutes? What about the other frailty? Severe frailty patients, just because they’re not […] dementia shouldn’t they get that same service?* PROF-13 (GP)

In practices where changes were successfully implemented, participants felt that patients and carers benefitted from enhanced care planning, particularly in the post-pandemic context:

*I think it’s been really useful for patients and relatives to have this intervention and understand that this is […] normal. You should be getting this.* PROF-05 (GP)

There was variability in stakeholder willingness or motivation to facilitate changes to the status quo of dementia care planning. Through this theme, we can consider *Contexts*, *Mechanisms* and *Outcomes*; the practical effects of implementation mechanisms. Some participants struggled with *negotiating capacity*, fitting the intervention within existing ways of working given contextual constraints. For some, there was clear *coherence*, with an understanding of potential benefits of enhanced care planning for patients (*internalisation*). Through *collective action*, diverse skillsets of the MDT could be harnessed (*skill-set workability*), leading to *relational restructuring* as the new roles and processes around care planning were formalised.

#### Theme 5: One size doesn’t fit all.

People with dementia and carers felt that current care provision was rarely tailored to their needs. In the broader context of dementia support, participants described some services as being unsuitable for their circumstances, particularly in cases where the person with dementia was younger than other attendees:

*C-03: I could see that he really wasn’t very comfortable in those surroundings. It’s difficult to put into words, but I think he felt that they were a lot worse off than – older than he is.*

*D-03: […] And what’s clouding all this as well is my severe deafness. I don’t pick up on what other people are saying.* C-03 (carer) and D-03 (person with dementia)

There was also an awareness that the needs of people with dementia and their carers were sometimes at odds, requiring increased sensitivity from healthcare professionals, or the opportunity for separate appointments.

People with dementia and carers commonly experienced a lack of meaningful information provision regarding their diagnosis or relevant services. Through the intervention, some participants reported improvements, with CDLs or practice staff highlighting available services.

Information provision alone was not sufficient to be meaningful or suggest personalisation of care. For example, a CDL missed an opportunity to personalise information provided to the needs of a family:

*Well we got a big envelope from [CDL]. We did get a big envelope but they’re just names and addresses and telephone numbers and I’ve got to say, I haven’t approached anyone.* C-14 (carer)

For many patients and carers, home visits were considered an act of personalisation, as well as staff taking time to get to know the individual’s circumstances. One carer spoke of the impact of a personal invite to a dementia one-stop-shop, with the GP assuring her that attending would be beneficial even without her husband:

*And actually, it encouraged me to go […] the fact that she said to me, “Why don’t you come along? I think it could be really useful,” in a really smiley [voice] that did encourage me to go, and think this is going to be useful, because she said it was going to be useful, and it was the first time I’d met her actually, she’s lovely [GP], what a lovely woman.* C-01 (carer)

Personalisation of care was valuable for people with dementia and carers. Although not all participants directly accessed the intervention, there was evidence of a move towards increased personalisation by practice staff and CDLs. Where care was viewed as personalised, this represented a meaningful change for participants.

#### Theme 6: Positive effects on people and systems: towards sustainability.

Many participants spoke positively about intervention benefits for practices and patients. Of the people with dementia and carers interviewed, some benefitted from direct and indirect effects of the intervention, although this was not the case for all. Most participants who attended annual dementia reviews found the experience beneficial, and hoped this would lead to sustained change:

*I thought it was a real, a great success. I hope they’ll do it sort of every year from now on…* D-02 (person with dementia).

One carer, however, reported a lack of follow-through following their attendance at a review:

*I went to the surgery ‘cause they had this thing for the dementia. […] I explained what I needed the help for, they took my details down, nobody’s called me.* C-05 (carer)

Where patients and carers had direct contact with the CDL, they were appreciative of the extra input and care received. For some it was hard to understand the service-level nature of the intervention; as such, not receiving support from the CDL was a source of disappointment.

Engaging entire staff teams in the intervention led to improved dementia-friendly practices; for example, embedding care alerts on electronic systems enabled reception staff to be aware that a patient may benefit from additional support; a change that was noted by patients and carers:

*I must admit that the receptionist or whoever it is that I’m calling who’s answering the phone, they seem to have improved to what they were before.* C-05 (carer)

Many professionals reported benefiting personally from the intervention in terms of increased knowledge and confidence regarding dementia care and services:

*We’ve grown in confidence supporting patients and carers with dementia. [CDL] has introduced us to some really good resources that we can access online, and also signposted us to different services […] she’s left us with knowledge that we’re able to carry on...* PROF-23 (Social prescriber)

The intervention also had an impact beyond patients and staff. Resources such as service mapping documents and dementia review templates were shared beyond the practices directly involved in the intervention, with CDLs using these resources and their experiences of the intervention to engage with local commissioners.

Many professionals felt changes were sustainable long-term, particularly those related to care planning. In some practices, staff had sought feedback from patients and carers to continue care planning improvements beyond the intervention. Questions about sustainability were raised by some participants. Some staff suggested that increasing the intervention duration may have increased the likelihood of changes being sustained. For others, benefits to patients and carers were central when considering longer-term, sustainable change to practice:

*I think just the value to patients and their carers of like, being heard, having time, having a chance to ask questions, trying to think ahead about the future. I think that that was so valuable to them. I suppose the question is, why wouldn’t we continue with it, rather than why did we decide to, that it just seemed to make logical sense.* PROF-06 (GP)

For many stakeholders, changes were viewed as desirable and sustainable, with positive impact for staff and patients alike. This theme can be considered in terms of implementation *Outcomes*. Changes made during the course of implementation continued following the close of the study (*intervention performance; sustainment*); specifically changes to care planning, referral systems through improved awareness of services, and dementia-friendly initiatives.

## Discussion

Previously we have reported on improvements to dementia care planning made through the PriDem intervention [20]. This paper has focused on the process of delivering this comprehensive, systems-level intervention in practice, specifically considering implementation barriers and facilitators that contributed towards or impeded its success.

Adaptation and flexibility of the intervention were key to delivery. Stakeholders were able to innovate and adapt within the intervention to better meet the needs of their service. This was most apparent in the development of multidisciplinary approaches to annual reviews, innovations within electronic records, and site-specific adaptations to PriDem resources.

Contextual factors were common across sites and impacted embedding of the intervention. Financial, capacity and staffing pressures were universal within primary care, exacerbated by the COVID-19 pandemic, with some staff questioning how enhanced dementia care could realistically be delivered within existing constraints. For some practices, this impacted negatively on engagement with key intervention components, thus limiting benefits for staff and patients. Future implementation studies may benefit from enhanced financial remuneration for participating sites and professionals to encourage engagement.

Globally, models have been implemented situating specialist staff within healthcare teams to improve dementia support [28,29], often focusing on direct patient care. CDLs found clinical work an effective means of building credibility within teams, encouraging engagement with and adoption of the intervention. However, overreliance on this component of the role compromised fidelity and acted as a barrier to implementation. In some cases, clinical work was prioritised over delivery of core intervention components. Although this served to ameliorate practice workload pressures, it suggests the intended role of the CDL and purpose of the intervention had been misunderstood. Greater clarity regarding role and intervention boundaries may have helped improve intervention fidelity. Through the application of NPT, we can see that some stakeholders struggled with *differentiation*, or understanding how the intervention was distinct from other services.

Findings do, however, illustrate mechanisms that can increase the likelihood of successful implementation within the primary care context. Motivated staff at all levels were able to encourage participation and drive change; particularly where intervention flexibility meant they were able to influence its direction, in line with existing literature on the role of champions in implementing healthcare innovations [30]. The identification of key stakeholders should be considered a priority for future implementation studies.

Unlike previous models of enhanced dementia support [28,29], a key focus of the PriDem intervention was the role of CDLs in upskilling generalists as a means of promoting staff capability. Stakeholders reported numerous positive outcomes of the intervention, including improved personalisation of care and communication with people with dementia and carers, staff confidence and knowledge of services and systems. Patients and carers benefitted from improvements to care planning processes, with many invited to attend an annual review for the first time. Despite contextual barriers, staff found workarounds to deliver improved services, particularly through engaging the broader MDT. NHS guidelines recommend up to 30 minutes for dementia care planning [31]; this level of contact may be facilitated through upskilling and utilising the wider workforce. Participants reported that skills developed during the intervention would have long-term impact on their approach to care provision, with changes made to processes perceived to be sustainable without ongoing CDL support.

### Strengths and limitations

This qualitative process evaluation benefits from inclusion of multiple data sources and a range of stakeholders. Observations and researcher fieldnotes provided insights into the process of implementation in real time. A wide range of professionals were interviewed, including roles underrepresented in primary care research (e.g., care coordinators, administrative staff). The study additionally benefitted from inclusion of people with dementia and carers in interviews, meaning indirect effects of the service-level intervention could be explored.

While it was important to include perspectives of people with dementia and carers, the service-level nature of the intervention was difficult to understand for some participants, particularly where they did not have direct exposure to intervention components. The sample for the feasibility study was weighted towards those with moderate to advanced cognitive impairment who were less likely to have decisional capacity, limiting the proportion eligible for interview. Despite these barriers, interviews provided valuable insights into the needs of this population, the reach of the intervention and its potential impact.

Analysis was strengthened by the involvement of multiple clinical and non-clinical members of the research team. Researchers from one region led the analysis. However, cross-site meetings took place throughout the analytic process to ensure a balanced perspective.

The design and analysis benefitted from the application of NPT. Rather than being constrained by its constructs, we found NPT was a valuable lens through which to view the data and make sense of the process of implementation. However, with its focus on intervention delivery, the model was not ideally suited to understanding the experiences of those intended to benefit from the intervention (people with dementia and carers). Despite this, through incorporating data from these participants we were able to represent these experiences in the analysis, including through the development of theme 5, ‘One size doesn’t fit all’, and relate them to the implementation findings.

A key concern of the study was the long-term sustainability of the intervention. Unfortunately, it was only possible to interview one professional post-intervention. As such, much of the data considers the likelihood of sustainment, rather than actual continuation of intervention components. Informally, the research team have tracked impact of the intervention, with evidence of continuation of changes to care planning, wider dissemination of study resources, and dementia customer care alerts still in operation. Future studies would benefit from extended follow-up periods to enable formal collection of longer-term data.

## Conclusions

Despite pressures facing primary care, meaningful change can be made to practice through a comprehensive, systems-level intervention. The presence of motivated and engaged staff are critical to implementation. Care needs to be taken to ensure understanding of complex interventions, so that fidelity is maintained. People with dementia and carers benefitted from improved care systems, with professionals considering changes made to be sustainable. Dementia service commissioners should consider the benefits of a CDL-led approach for practices, staff and patients.

## Supporting information

S1 File Standards for Reporting Qualitative Research (SRQR) checklist. (DOCX)
